# Functional changes in mRNA expression and alternative pre-mRNA splicing associated with the effects of nutrition on apoptosis and spermatogenesis in the adult testis

**DOI:** 10.1186/s12864-016-3385-8

**Published:** 2017-01-10

**Authors:** Yongjuan Guan, Guanxiang Liang, Graeme B. Martin, Le Luo Guan

**Affiliations:** 1 UWA Institute of Agriculture and School of Animal Biology, University of Western Australia, 35 Stirling Highway, Crawley, WA 6009 Australia; 2 Department of Agricultural, Food and Nutritional Science, University of Alberta, Edmonton, AB T6G 2P5 Canada; 3 Present address: 304 Rosenthal, 3800 Spruce Street, Philadelphia, PA 19104 USA

**Keywords:** Nutrition, Spermatogenesis, Apoptosis, mRNAs, Pre-mRNA alternative splicing

## Abstract

**Background:**

The effects of nutrition on testis mass in the sexually mature male have long been known, however, the cellular and molecular processes of the testis response to nutrition was not fully understood.

**Methods:**

We tested whether the defects in spermatogenesis and increases in germ cell apoptosis in the testis that are induced by under-nutrition are associated with changes in mRNA expression and pre-mRNA alternative splicing using groups of 8 male sheep fed for a 10% increase or 10% decrease in body mass over 65 days.

**Results:**

We identified 2,243 mRNAs, including *TP53* and *Claudin 11*, that were differentially expressed in testis from underfed and well-fed sheep (FDR < 0.1), and found that their expression changed in parallel with variations in germ cell numbers, testis size, and spermatogenesis. Furthermore, pairs of 269 mRNAs and 48 miRNAs were identified on the basis of target prediction. The regulatory effect of miRNAs on mRNA expression, in combination with functional analysis, suggests that these miRNAs are involved in abnormal reproductive morphology, apoptosis and male infertility. Nutrition did not affect the total number of alternative splicing events, but affected 206 alternative splicing events. A total of 159 genes, including *CREM*, *SPATA6*, and *DDX4*, were differentially spliced between dietary treatments, with functions related to RNA splicing and spermatogenesis. In addition, three gene modules were positively correlated with spermatogenesis-related phenotypic traits and negatively related to apoptosis-related phenotypic traits. Among these gene modules, seven (*CFLAR*, *PTPRC*, *F2R*, *MAP3K1*, *EPHA7*, *APP, BCAP31*) were also differentially expressed between nutritional treatments, indicating their potential as markers of spermatogenesis or apoptosis.

**Conclusions:**

Our findings on significant changes in mRNAs and pre-mRNA alternative splicing under-nutrition suggest that they may partly explain the disruption of spermatogenesis and the increase germ cell apoptosis. However, more research is required to verify their causal effects in regulating spermatogenesis and germ cell apoptosis.

**Electronic supplementary material:**

The online version of this article (doi:10.1186/s12864-016-3385-8) contains supplementary material, which is available to authorized users.

## Background

The development of mature haploid spermatozoa from diploid spermatogonial cells [1] can be affected by many factors, including photoperiod, hormones, temperature and nutrition. The effects of nutrition on testis mass in the sexually mature male have long been known, as has the direct relationship between testicular mass and sperm production [2]. In addition, with change in testicular size, the efficiency of sperm production also changes [3]. We have been investigating the cellular and molecular processes of the testis response to nutrition, and we have found that under-nutrition despaired spermatogenesis in adult sheep [4, 5].

Within the testis, spermatogenesis is a strictly regulated process, at both the transcriptional and the post-transcriptional level [6]. In recent years, a novel mechanism of post-transcriptional control, mediated by microRNAs (miRNAs), has emerged as an important regulator of spermatogenesis [6, 7]. miRNAs are small (~22 nucleotides) endogenous RNAs that negatively regulate gene expression by targeting the 3′untranslated region (3′UTR) [8] and/or coding region [9] of mRNAs. We have recently found that the expression of a number of miRNAs is affected by nutrition in sexually mature male sheep, and most of the predicted targets of the differentially expressed miRNAs were mainly involved in reproductive system development and function [10]. However, the regulatory relationship between these miRNAs and their corresponding mRNAs targets in testis remains to be verified. We therefore decided to profile mRNA expression in the testes of well-fed and underfed male sheep using RNA-seq so we could explore the relationships between the miRNAs we had identified and their putative targets.

In addition to the disruption of spermatogenesis, under-nutrition of sexually mature male sheep increased apoptosis in germ cells [10]. Our recent study has revealed higher levels of expression of miR-98 in underfed sheep than in well-fed sheep [10] and this miRNA has been reported to play a critical role in apoptosis [11]. Since the molecular mechanisms through which miRNAs regulate the expression of apoptosis-related genes are still controversial, we decided to explore these processes further using our nutritional model.

It has also been reported that spermatogenesis and a large number of apoptotic factors are regulated by alternative pre-mRNA splicing that generates multiple transcript species from a common mRNA precursor and thus raises protein diversity and allows the system to cope with the increasingly broad spectrum of functional and behavioural complexity [12, 13]. To date, eight types of alternative splicing have been reported: cassette exon, alternative 5′ splice site, alternative 3′ splice site, mutually exclusive exon, coordinates cassette exons, alternative first exon, alternative last exon and intron retention [14]. We therefore also tested the hypothesis that nutritional treatment will induce differences in alternative splicing, and these changes will be related to the regulation of spermatogenesis and germ cell apoptosis in the testis.

Overall, this study used testicular tissue from under-fed and well-fed sexually mature sheep to pursue four objectives: 1) To investigate the differences of the expression of mRNAs; 2) To evaluate the influence of miRNAs on spermatogenesis and the expression of apoptosis-related genes; 3) To explore the relationships between alternative pre-mRNA splicing and spermatogenesis and apoptosis; 4) To investigate the relationships between the gene modules and spermatogenesis and apoptosis related phenotypic traits.

## Methods

The experimental protocol was approved by the Animal Ethics Committee of the CSIRO Centre for Environment and Life Sciences, Floreat, Western Australia (Project No.1202).

### Animals and treatments

From May to July (autumn-winter), 24 Merino male sheep (age 24 months, body mass 65.7 ± 4.7 kg, scrotal circumference 31.8 ± 2.5 cm) were housed in individual pens in a building with windows that allowed good penetration of natural light at Floreat, Western Australia. During the 3-week acclimatization period, all sheep were fed daily with 750 g oaten chaff (8.4% crude protein; 8.0 MJ/Kg metabolisable energy) and 200 g lupin grain (35.8% crude protein; 13.0 MJ/Kg metabolisable energy). At the start of the treatment period (end of May; mid-autumn), the animals were allocated into three dietary treatment groups (high, maintenance and low) balanced for training success to semen collection, body mass, scrotal circumference, temperament, poll-horn type, and sperm quality (the percentage of live and motile sperm, sperm concentration). Animals fed their maintenance requirements were expected to maintain constant body mass. The high diet was designed to allow the animals to gain 10% live weight over 65 days whereas the low diet was designed to allow 10% loss in weight. At the start of the treatment period, individual daily allowance was 1.2 kg oaten chaff plus 0.3 kg lupin grain for the rams in the high-diet group, 0.7 kg chaff and 0.18 kg lupin grain for the maintenance group, and 0.51 kg chaff and 0.13 kg lupin grain for the low-diet group. Every week, the animals were weighed and the amount of feed offered to each individual was adjusted to ensure achievement of target live weight.

The data on the effects of nutritional treatments on body mass, scrotal circumference, semen quality and spermatozoal quality, Sertoli cell number and quality were published before [4, 5]. Our previous work showed significant differences between High diet and Low diet in terms of the above parameters, with the values for maintenance-fed rams being generally similar to those for the High diet. For purposes of the current study, we decided to perform RNA-Seq only with the two extreme groups (testis growing versus testis shrinking) to determine the factors that contribute to the huge differences in phenotype.

### Tissue Collection and preservation

After 65 days, all male sheep were killed with intravenous overdose of sodium pentobarbitone, and the testes were immediately removed, dissected and weighed. Three samples were chosen from top, middle and bottom parts of both testes (~1 cm^3^ for each); those from the right testis were snap-frozen in liquid nitrogen and stored at −80 °C for RNA isolation.

### Isolation of RNA

About 1 cm^3^ tissues from top, middle and bottom parts of the right testes were mixed and grinded to powder in liquid nitrogen for RNA isolation. The trizol method was used to isolate total RNA [15] from testis samples. The quality and quantity of the RNA were determined by Agilent 2100 Bioanalyzer (Agilent Technologies, Santa Clara, CA) and Qubit 2.0 Fluorometer (Invitrogen, Carlsbad, CA). Only RNA with an integrity number (RIN) greater than 7.0 was used for further analysis.

### Small RNA library sequencing and Identification of miRNAs

The methodology details and outcomes for miRNAs have been reported elsewhere [10].

### Construction and sequencing of the RNA-seq library

Total RNA (1.0 μg each) from each sample was used to construct RNA-seq libraries with a unique index using the TruSeq mRNA Sample Preparation kit (Illumina, San Diego, CA) according to the manufacturer’s instruction. Quantitative real time PCR (qPCR) was performed for library quantification using the StepOnePlus™ Real-Time PCR System (Applied Biosystems, Carlsbad, CA) and KAPA SYBR Fast ABI Prism qPCR kit (Kapa Biosystems, Woburn, MA). Individual libraries were then pooled for sequencing at Génome Québec (Montréal, Canada) using the HiSeq 2000 system (Illumina). Sequencing was performed as 100 bp paired-end reads. All reads were de-multiplexed according to their index sequences with CASAVA version 1.8 (Illumina) and reads that did not pass the Illumina chastity filter were discarded.

### Mapping and annotation of RNA-seq reads

RNA-seq reads were aligned to the ovine genome (OAR 3.1) using Tophat 2.0.10 with default parameters [16]. Each BAM file obtained from TopHat2 and the GTF file obtained from ENSEMBL (v75.30) were used in the htseq-count (http://www-huber.embl.de/users/anders/HTSeq/) to determine the number of reads mapped to each gene.

### Identification of differentially expressed (DE) mRNAs

Differentially expressed (DE) mRNAs were investigated with the bioinformatics tool, edgeR that utilizes a negative binomial distribution to model sequencing data [17]. The expression of mRNAs in each library was normalized to counts per million reads (CPM) with the formula: CPM = (reads number/total reads number per library) × 1,000,000. mRNAs with CPM > 5 in at least 50% of the samples were subjected to DE analysis. Fold changes were defined as ratios of arithmetic means of CPM within each comparison group. Significant DE mRNAs were determined by an adjusted *P* value (False discovery rate, FDR < 0.1) based on Benjamini and Hochberg multiple testing correction [18] as well as fold change > 1.5 [19].

### Validation of mRNA expression using RT-qPCR

RT-qPCR was performed using SYBR Green (Fast SYBR® Green Master Mix; Applied Biosystems) to validate mRNA expression of six differentially expressed genes: *PIWIL1*, *SPATA4, INHBA*, *FOXO3*, *PTEN*, *CYP51A1*. Oligonucleotide primer sequences for these genes were designed using NCBI primer blast (http://www.ncbi.nlm.nih.gov/tools/primer-blast/index.cgi?LINK_LOC=BlastHome) and the primer for glyceraldehyde 3-phosphate dehydrogenase (GAPDH) was obtained from a published source (Table 1). Total RNA (1 μg) from each sample was treated with DNAase I (Invitrogen), and reverse-transcribed to cDNA using SuperScript II reverse transcriptase following the manufacturer’s protocol (Invitrogen). Fluorescence signal was detected with StepOnePlus™ Real-Time PCR System (Applied Biosystems). In total, each reaction contained 10 μl Fast SYBR Green Master Mix (Applied Biosystems), 1 μl of forward primer (20 pmol/μl), 1 μl of reverse primer (20 pmol/μl), 7 μl nuclease-free water, and 1 μl cDNA template. Samples were measured in triplicate using the following protocol: 95 °C for 10 min for initial denaturation and then 40 cycles of 95 °C for 20 s, followed by annealing/extension for 30 s at 60 °C. Analysis of melting curves was used to monitor PCR product purity. Amplification of a single PCR product was confirmed by agarose gel electrophoresis and DNA sequencing (data not shown). One-way ANOVA was used to compare the groups, and *P* < 0.05 was considered significant. Data are expressed as Mean ± SEM.

**Table 1:** Details of primers used for RT-qPCR

Gene	Abbrev.	Sequence	Product length (bp)
Piwi-Like RNA-Mediated Gene Silencing 1	PIWIL1	F:CTGGTTCTCTCGCTGTGTGT	90
		R:TTCCAAGCCCTTAGAGCAGC	
Spermatogenesis Associated 4	SPATA4	F:CTCTCGATCACCATCCTGCC	106
		R:CTCAATGCACTCATGCTCGC	
Inhibin beta A	INHBA	F:AGGTGGTGGATGCTCGAAAG	125
		R:GGTCTCCTGACACTGCTCAC	
Forkhead box O3	FOXO3	F:ATGGCAACCAGACACTCCAG	97
		R:CTGGCCTGAGACATCAAGGG	
Phosphatase and tensin homolog	PTEN	F:CGGCCGTTCCGAGGATT	99
		R:CTGGATGGTTGCAGCGACT	
Cytochrome P450, Family 51, Subfamily A, Polypeptide 1	CYP51A1	F:TACCTACCTGCTGGGGAGTG	107
		R:TCCCAAACACAGGTGTCGTC	
Glyceraldehyde-3-phosphate dehydrogenase	GAPDH	F:CTGCTGACGCTCCCATGTTTGT	150
		R:TAAGTCCCTCCACGATGCCAAA	

### Construction of miRNA-mRNA regulatory relationships

The results for miRNAs were all obtained from a previous study using the same samples [10]. The predicted regulatory relationships between differentially expressed miRNAs and differentially expressed mRNAs were identified on the basis of two criteria as suggested by previous studies [20, 21]: negative correlation and computational target prediction. Genes targeted by miRNAs were predicted by TargetScan Release 6.0 (http://www.targetscan.org/) and miRanda (http://www.microrna.org/microrna/home.do). Target genes predicted by both TargetScan (default parameters) and miRanda (total score > 145, total energy < −10 kcal/mol) were used for further analysis [22]. Pairwise Pearson correlation coefficient (R) was computed for each miRNA and their predicted target genes, and multiple testing corrections were done by calculating FDR. Significant miRNA-mRNA pairs were defined as R < −0.9 and FDR < 0.1.

### Functional analysis for DE mRNAs

The identities of the DE mRNAs in the miRNA-mRNA regulatory relationships were uploaded into IPA software (Ingenuity Systems, www.ingenuity.com) to detect the top functions. A threshold of *P* < 0.01 was applied to enrich significant biological functions. The IPA regulation *z*-score algorithm was used to predict the direction of change for a given function (increase or decrease), with a *z*-score > 2 suggesting a significant increase whereas a *z*-score < −2 suggesting a significant decrease. The GO terms were defined and the KEGG pathways were enriched using Database for Annotation, Visualization and Integrated Discovery (DAVID, http://david.abcc.ncifcrf.gov) [23]. For each analysis, the functional annotation clustering option was used and significant GO terms and KEGG pathways were declared at *P* < 0.05 and molecule number > 2.

### Identification and annotation of alternative splicing (AS) events

TopHat2 was used to predict the splice junctions (options: −a 19, −g 1, −-max-intron-length 17325, and --min-intron-length 81). A total of 15 million reads were randomly selected from each sample for analysis to make sure that the comparison was at the same level [14]. JuncBASE was used to annotate all AS events (cassette exons, alternative 5′ splice site, alternative 3′ splice site, mutually exclusive exons, coordinate cassette exons, alternative first exons, alternative last exons, and intron retention) and calculate the Percentage Spliced Index (PSI) [24]. Splicing analysis was performed for events that had at least 20 reads and the PSI differences (ΔPSI) are higher than 10% [25].

### The relationship between gene modules with sheep phenotypic traits

A weighted gene co-expression network was constructed for all samples using the WGCNA package in R to analyse the expressed mRNAs (CPM > 1 in at least 8 samples) [26]. Briefly, a matrix of pairwise correlations between all pairs of genes across all samples was constructed. An adjacency matrix was then calculated, using the correlation matrix of the expression sets, and transformed into a topological overlap matrix that was then used to derive a distance matrix of hierarchical clustering. Finally, the mRNAs were assigned into different modules based on hierarchical clustering. Modules with eigengenes (defined as the first principal component of each module and considered as a representative of the gene expression profiles in that module) that were highly correlated were then merged. All these steps were performed using the ‘blockwiseModules’ function in the WGCNA package, using the major parameters described previously [27]. The correlations were calculated for the relationships between module eigengenes and phenotypes (testis weight, sperm number per testis, tubule diameter, seminiferous epithelium volume, change of scrotal circumference, apoptotic cells/tubule; Additional file 1: Table S1).

## Results

### High Quality RNA-seq data were obtained from all samples

**Fig. 1:**
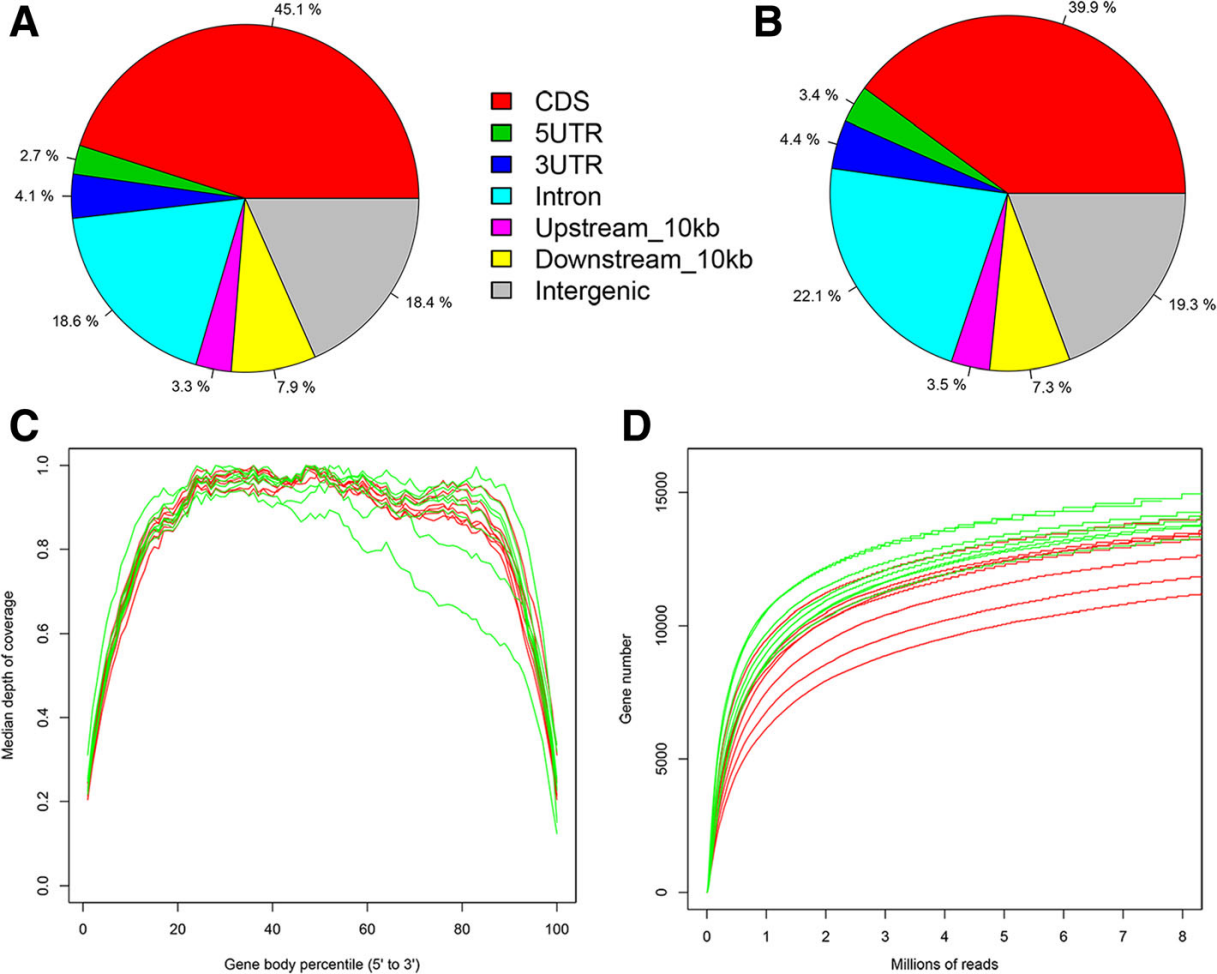
Quality and features of the RNA-seq datasets obtained from testis of male sheep. Distribution of the RNA-seq reads from High Diet group (**a**) and Low Diet group (**b**) along the annotated features of the sheep genome. **c** Relationship between the RNA-seq read coverage and the length of the transcriptional unit. The x-axis indicates the relative length of the transcripts. **d** Saturation curve for gene detection. Randomly sampled reads are plotted against the expressed genes

More than 350 million sequenced paired-end reads were obtained from 16 libraries, of which an average of 76% could be mapped to OAR3.1 (http://www.livestockgenomics.csiro.au/). The genomics region of reads, the RNA-seq 3′/5′ bias and the sequencing depth were analysed to evaluate the quality of the RNA-seq data. Around 50% of the reads were derived from exonic regions, while around 20% were derived from intergenic or intronic regions (Fig. 1a and b). In general, the coverage of reads along each transcript revealed no obvious 3′/5′ bias (Fig. 1c). As can be seen in Fig. 1d, the number of transcripts detected increased as the number of the sequencing reads increased. Finally, analysis of the sequencing coverage along each chromosome showed extensive transcriptional activity for the entire genome (Additional file 2: Figure S1).

### Profile of mRNAs in sheep testis

An average of 13,980,416 (SD = 2,930,788) reads from high diet and 11,014,809 (SD = 2,524,631) from low diet were mapped to Ensembl gene annotation database (*P* < 0.05). A total of 13,859 genes were detected in testicular tissue from the low diet group, compared to 14,561 from the high diet group. In total, 11,748 genes were expressed in all 16 animals. The most abundant transcript (~2% of total reads) was from the 7SK gene, a small nuclear RNA involved in pre-mRNA splicing and processing. Functional analysis with DAVID software revealed that the most highly expressed 3,000 genes were mainly related to cell cycles, protein catabolic processes, and spermatogenesis (Table 2). Only genes (14,385) that were expressed in at least 8 libraries were used for further analysis.

**Table 2:** The 15 functions most commonly related to the most highly expressed 3000 genes (as determined by DAVID software). *P* value indicates the relevance of the function (lower value means greater relevance)

Term	Count	*P*-Value
cell cycle	232	5.49E-35
cell cycle process	185	2.43E-33
modification-dependent macromolecule catabolic process	177	2.43E-28
modification-dependent protein catabolic process	177	2.43E-28
proteolysis involved in cellular protein catabolic process	181	1.05E-27
cellular protein catabolic process	181	2.03E-27
protein catabolic process	184	4.97E-27
M phase	121	7.18E-27
intracellular transport	190	1.39E-26
male gamete generation	114	1.30E-25
spermatogenesis	114	1.30E-25
cellular macromolecule catabolic process	200	3.66E-25
cell cycle phase	135	3.62E-24
macromolecule catabolic process	208	4.51E-24
sexual reproduction	144	5.26E-24

### Effects of nutrition on mRNA expression

**Fig. 2:**
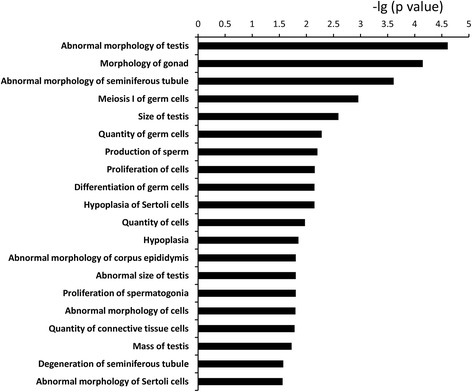
The 20 functions most commonly related to the differentially expressed mRNAs in testis from underfed and well-fed male sheep, as determined by IPA software. *P* value indicates the relevance of the function (lower value means greater relevance)

In total, 2,243 mRNAs were found to be differentially expressed (DE) when comparing underfed with well-fed male sheep (Additional file 3: Table S2), of which 1,081 were expressed more in underfed than well-fed sheep (eg, *TP53* and *Claudin 11*) and 1,162 were expressed less in underfed than well-fed sheep (eg, *CYP51A1* and *SPATA4*)*.* IPA analysis revealed that the functions of most of the DE *mRNAs* are related to quantity of germ cells, testis size, quantity of Sertoli cells, and quantity of connective tissue cells (Fig. 2). We considered genes that were related to more than one function may be more important, such as FOXO3, PTEN, CYP51A1, INHBA and SPATA4. Therefore, they were selected for further RT-qPCR validation analysis. Further functional analysis using DAVID, produced largely the same outcome, indicating that most common functions of DE mRNAs to be in the cell cycle (*n* = 116), spermatid development (*n* = 16), spermatogenesis (*n* = 48), and DNA replication (*n* = 18) (Additional file 4: Table S3). Importantly, one gene, *PIWIL1* (*MIWI*), were associated with all of these functions.

### RT-qPCR validation of differentially expressed genes

Six mRNAs were selected from the DE mRNAs and the RT-qPCR results were consistent with the sequencing data for all of them. For example, both the sequencing data and the RT-qPCR results showed that *PIWIL1* was expressed at a lower level in underfed males than in well-fed males (Additional file 5: Figure S2). In addition, the expression of *INHBA* was down regulated in well-fed male sheep (Additional file 5: Figure S2).

### The relationship between gene modules with sheep phenotypic traits

**Fig. 3:**
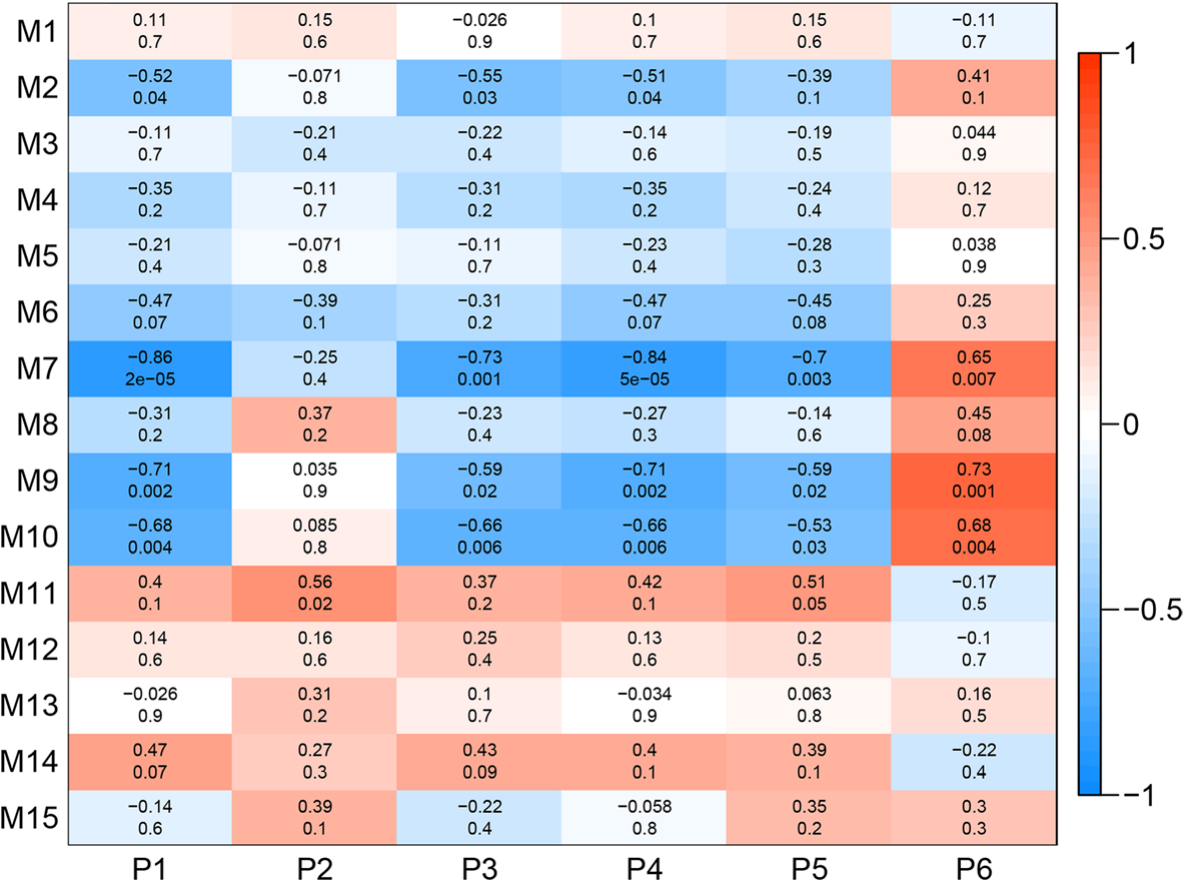
The relationships between gene modules and phenotypic traits in the testis of mature sheep. The y-axis shows the 15 gene modules constructed by weighted gene co-expression network analysis (WGCNA). The x-axis shows the phenotypic traits: P1 indicates testis weight (g); P2 indicates sperm number per testis; P3 indicates diameter of seminiferous tubule; P4 indicates volume of seminiferous epithelium (x 10^12^ μm^3^); P5 indicates change of scrotal circumference; P6 indicates apoptotic germ cells/tubule. In each cell of the table, the upper value shows the coefficient of correlation (r) between gene module and phenotypic trait, while the lower value indicates the statistical probability. Red to blue colouration of the cells indicates the transition from positive to negative correlation, as indicated by the colour bar

A total of 15 modules were obtained using WGCNA analysis, of which three (Modules 7, 9 and 10) were of interest because the relationships were strong (correlation coefficient > ± 0.5; *P* < 0.01). These modules were negatively correlated with testis weight, tubule diameter, seminiferous epithelium volume and change in scrotal circumference, and positively correlated with apoptotic cells/tubule. Among the 15 modules, only Module 11 was correlated with sperm number per testis (*r* = 0.56, *P* = 0.02; Fig. 3).

### Functional analysis for the genes in Modules 7, 9 and 10

Functional analysis suggested that genes in Modules 7 and 9 were associated with both spermatogenesis and apoptosis, but genes in Module 10 were only related to apoptosis. Specifically, in Module 7, 35 genes were related to spermatogenesis whereas 46 genes were associated with apoptosis. Six genes (*NFKBIL1*, *XRCC5*, *ERCC1*, *APP*, *BCAP31, RRAGA*) were related to both spermatogenesis and apoptosis. For Module 9, eight genes (*RXFP1*, *ITCH*, *ITGB1*, *XHD*, *TAF7L*, *WNT2*, *SPIN1, LNPEP*) were associated with spermatogenesis, whereas 16 genes (*CFLAR*, *TAF9B*, *CCK*, *VAV3*, *NR3C1*, *CDH13*, *CROP*, *CDKN1B*, *PTPRC*, *ATP7A*, *RTN3*, *HSPD1*, *ITM2B*, *F2R*, *MAP3K1, RAD21*) were associated with apoptosis, but no genes were common to spermatogenesis and apoptosis. For Module 10, four genes (*EPHA7*, *SCIN*, *NGFRAP1* and *MAP3K7*) were related to apoptosis. Interestingly, of all the genes mentioned above, seven (*APP*, *BCAP31*, *CFLAR*, *PTPRC*, *F2R*, *MAP3K1*and *EPHA7*) were differentially expressed between nutritional treatments, indicating their pivotal roles in reproduction or apoptosis.

### miRNA-mRNA regulatory relationships

**Fig. 4:**
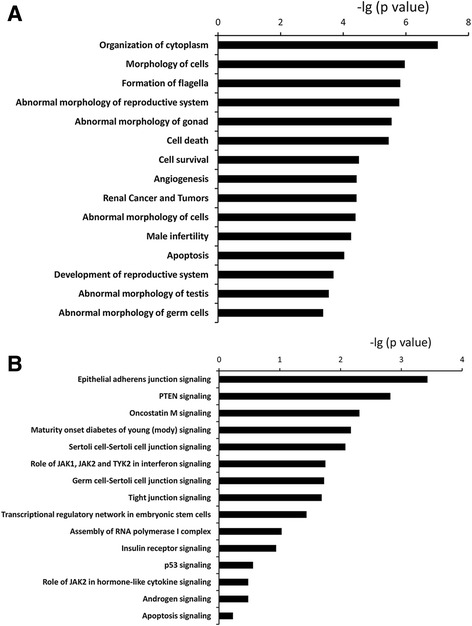
The 15 functions (**a**) and the 15 signalling pathways (**b**) most commonly related to the mRNAs in miRNA-mRNA regulatory relationships in sheep testis, as determined by IPA software. *P* value indicates the relevance of the function (lower value means greater relevance)

Putative miRNA-mRNA pairs were identified on the basis of target prediction and the negative regulatory effect of miRNAs on the expression levels of their target genes. A total of 940 miRNA-mRNA pairs (48 miRNAs, 269 mRNAs) were identified (Additional file 6: Table S4). Among these pairs, oar-novel-miR-33 and oar-novel-miR-31 paired with the highest number of mRNAs: oar-novel-miR-33 paired with 68 mRNAs and oar-novel-miR-31 paired with 52 mRNAs (Additional file 7: Figure S3). IPA analysis indicated that the mRNAs in the negative pairs were mainly involved with organization of cytoplasm, cell morphology, abnormal morphology of the reproductive system, cell death and male infertility (Fig. 4a). In addition, these mRNAs were also involved in 76 signalling pathways, of which Sertoli cell-Sertoli cell junction signalling, germ cell-Sertoli cell junction signalling, androgen signalling, and apoptosis signalling were among the 15 most relevant (Fig. 4b). FOXO3 and PTEN were related to more than 8 functions out of 15 most common functions, we assumed these two genes may be crucial for testes function, therefore, they were selected for RT-qPCR validation. The expression of FOXO3 was higher in well-fed sheep than underfed sheep, while PTEN expression was lower in well-fed than underfed sheep.

### Identification of alternative splicing events

We initially obtained 940,607 junctions from the 16 RNA-seq libraries with the Tophat2 software. Totally, 6376 alternative splicing events (∆PSI > 10% in at least one library and at least 20 reads mapped) were identified from these junctions (Additional file 8: Table S5). Among these alternative splicing events, 4,820 (75.6%) were previously annotated as known alternative splicing events in the Ensembl Database, which can be mapped to 2288 unique genes. Eight types of alternative splicing events were identified, including 1,131 cassette exons, 645 alternative 5′ splice sites, 578 alternative 3′ splice sites, 17 mutually exclusive exons, 86 coordinate cassette exons, 578 alternative first exons, 247 alternative last exons, and 2,810 intron retentions.

### Effects of nutrition on alternative splicing

**Fig. 5:**
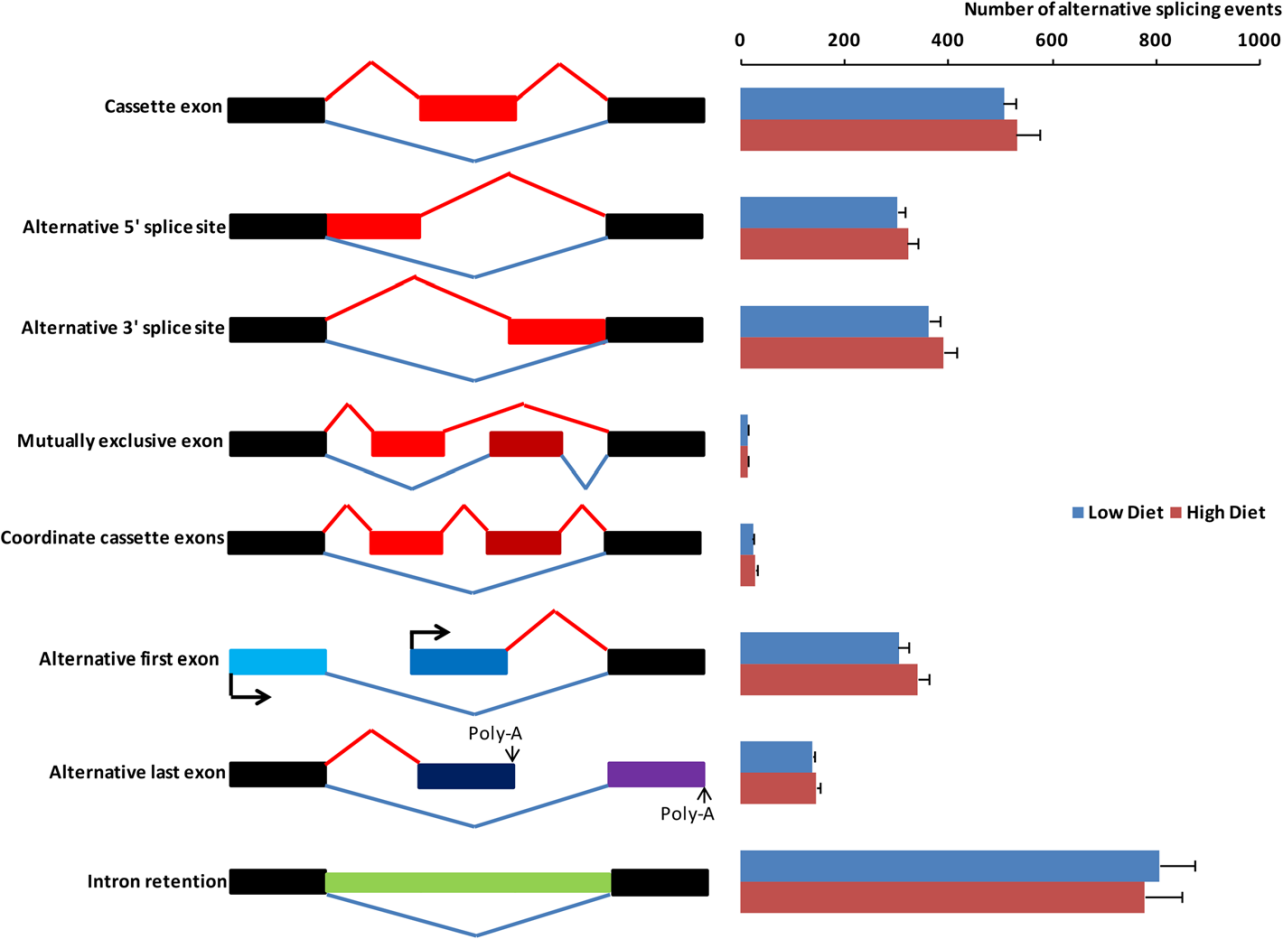
The counts of each type of alternative splicing event in testicular tissues from underfed and well-fed male sheep. Values = Mean ± SEM (*n* = 8). Most common alternative splicing events were cassette exon, intron retention, alternative first exon and alternative last exon. On the other hand, four types were not as common, they were alternative 3′ splice site, mutually exclusive exons, alternative 5′ splice site, coordinate cassette exons

**Fig. 6:**
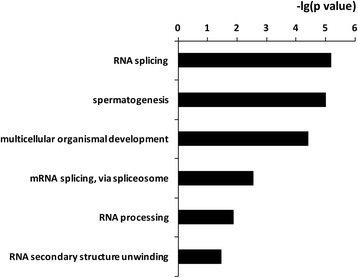
The top functions most commonly related to differential alternative splicing events in testis from sheep fed a high diet or a low diet. *P* value indicates the relevance of the function (lower value means greater relevance)

We found 2551 ± 189 (Mean ± SEM) alternative splicing events in the High Diet group and 2455 ± 126 alternative splicing events in the Low Diet group (not significant). There was no difference as for the total number of each type of alternative splicing event between two groups (Fig. 5). PSI values from each diet group were used to test the differentially spliced genes between treatments, resulting 206 differentially spliced isoforms (21 alternative 3′ splice sites, 23 alternative 5′ splice sites, 34 alternative first exon, 17 alternative last exon, 86 cassette, 7 coordinate cassette exon, 8 intron retention events, *P* < 0.05, Wilcoxon test). These differentially spliced isoforms were mapped to 159 known unique genes, and DAVID functional analysis revealed the most common functions of these genes were related to RNA splicing and spermatogenesis (Fig. 6).

## Discussion

This study appears to be the first to profile the whole transcriptome in sheep testis, to construct the regulatory relationships between miRNAs and mRNAs in testis, to explore the relationships between pre-mRNA alternative splicing and testis function, and to link the gene modules with phenotypic traits related to spermatogenesis and apoptosis. In the context of an experimental model of reversible testis growth in the sexually mature male, we have been able to identify mRNAs that are associated with testis function and, more importantly, apoptosis in germ cells. These findings strongly support the hypothesis that the decline in spermatogenesis and increase in germ cell apoptosis induced by under-nutrition in the sexually mature male sheep are, at least, partially due to changes in mRNA expression and pre-mRNA alternative splicing.

We found over 2,000 mRNAs that were differentially expressed between treatments, with over 1,000 mRNAs, including *TP53* and *Claudin 11*, that were more highly expressed in underfed than in well-fed sheep. This result supports our previous observations based on qPCR [5]. A high level of *TP53* indicates more cells going through apoptosis [28], and this result is in agreement with our previous finding-more TUNEL positive cells were observed in testes from underfed than in well-fed sheep [10], so we conclude that under-nutrition increases apoptosis in germ cells. On the other hand, *Claudin-11* is a tight junction protein expressed in Sertoli cells and rarely in other cell types in the testis [29] and plays a central role in the formation of tight junctions [30, 31]. In testicular tissue from underfed sheep, increased expression of *Claudin 11* and disorganization of Claudin 11 protein strongly indicate the impairment of tight junctions [5]. In addition, in the present study, over 1000 mRNAs showed lower expression in underfed than in well-fed sheep, including *CYP51A1* and *SPATA4*. CYP51A1 is a member of the cytochrome P450 family and is expressed strongly by germ cells [32], illustrating its crucial role in spermatogenesis. Therefore, the lower level of *CYP51A1* expression in underfed sheep is coherent with the decrease in numbers of germ cells and defective spermatogenesis caused by undernutrition [10]. *SPATA4* has also been reported to be testis-specific and associated with spermatogenesis, and involved in maintaining spermatogenesis [33]. Therefore, the reduced expression of *SPATA4* in underfed sheep is also consistent with compromised spermatogenesis with under-nutrition.

The functional analysis using IPA revealed that, for mRNAs that are differentially expressed between nutritional treatments, the most common functions are quantity of germ cells, testis size, quantity of Sertoli cells and quantity of connective tissue cells. Thus, the differentially expressed transcriptomes are consistent with the reductions of testis mass and sperm production in underfed rams [10]. Among all the genes that were related to these functions, we considered the genes that were related to more than one function may be more important, such as *IGF1R*, *INHBA*, *TP53*. In the testes of adult mice lacking *IGF1R* in their Sertoli cells, there is a reduction in testis size and daily sperm production [34], indicating a role for *IGF1R* in control of sperm production by Sertoli cells. A protein product of the *INHBA* gene, activin A, is an important regulator of testicular cell proliferation [35]. As indicated above, *TP53* regulates spermatogenesis by inducing apoptosis [36]. We propose that, the expression of *IGF1R, INHBA* and *TP53* may be used as biomarkers of sperm production.

To further investigate the crucial genes in spermatogenesis and apoptosis, we looked at the relationships between these phenotypes and the gene modules that we discovered in the testis. We found five genes (*CFLAR*, *PTPRC*, *F2R*, *MAP3K1*, *EPHA7*) that appear to be crucial for apoptosis and two genes (*APP* and *BCAP31*) related to both apoptosis and spermatogenesis. More importantly, all seven of these genes were differentially expressed between nutritional treatments, suggesting that they played pivotal roles in the control of testis function. These conclusions are supported by previous studies. For example, *CFLAR* is involved in inhibition of the death receptor-activated pathway [37]; *MAP3K1* has both anti- and pro-apoptotic functions [38]; *EPHRINA5*-*EPHA7* complex induces apoptosis through *TNFR1* [39]. Therefore, these seven genes are potential biomarkers of spermatogenesis and apoptosis. Interestingly, the changes in these genes were associated with change in testis mass, raising the possibility that factors associated with change in testicular tissue, rather than direct effects of nutritional treatments on testicular tissue, are responsible for changes in spermatogenesis and apoptosis [5, 10]. If this were to be the case, then the relationships between gene modules and phenotypes observed in the present study could be applied more generally to other factors that can cause changes in the testis mass, such as photoperiod, stress and temperament, or physical fitness.

In addition, this study further identified miRNA-RNA relationships which may regulate the above altered expression events. Of particular importance are oar-novel-miR-33 and oar-novel-miR-31 because they paired with the greatest number of mRNAs, indicating a crucial role in testis function. Novel-miR-33 is homologous to miR-296 that is specific to embryonic stem cells and has been reported to be highly conserved between species [40]. So far, there is no direct evidence for a role for miR-296 in testis function. However, one study proved that miR-296 was more highly expressed in mature testis than in immature testis, indicating a pivotal role in spermatogenesis in the adult. In addition, miR-296 was also defined as anti-apoptotic [41]. Therefore, the reduced expression of novel-miR-33 (miR-296) in underfed sheep [10] illustrates decreased testis function and increased cell apoptosis. By contrast, novel-miR-31 is homologous with miR-34 which has been shown to enhance germ cell phenotype during the late stages of spermatogenesis in other species [42]. In the current study, therefore, the lower level of novel-miR-31 in underfed sheep is coherent with the loss of germ cell function [10].

Nutritional treatment did not affect the total number of alternative splicing junctions, in contrast with some previous reports of nutritional effects on other biological processes [43, 44]. The lack of effect of nutritional treatment on the total number of alternative splicing junctions suggests that alternative splicing is a fine-tuner in the testis that stabilizes testis function, as suggested for other tissues [45, 46]. However, with respect to specific genes, we found 159 that were differentially spliced between high diet and low diet groups. Functional analysis of these genes indicated roles in RNA splicing and spermatogenesis, and suggests that nutrition affects spermatogenesis by changing pre-mRNA splicing. For example, the alternative splicing event for *CREM* (cAMP response element modulator) is alternative last exon. It has been reported that *CREM* mRNA exhibits a remarkable array of alternative splice variants [47]. For example, during male meiosis, the inactive *CREM* variant is switched to active *CREM* variant (by incorporation of transactivating domains) directed by alternative splicing. Therefore, in the mature male sheep, it is possible that nutrition affects the number of active *CREM* isoforms, potentially explaining the disruption of spermatogenesis in underfed sheep. Future studies on verification of alternative splicing events genes related to spermatogenesis detected by RNA-seq using qPCR and how such changes affect the activity, possibly involving construction of a shortened ‘minigene’ containing the regulated exons and splicing signals [48], will be required to better understand this process. In addition, it is essential to determine whether the changes in splicing can affect protein expression.

## Conclusions

In conclusion, we have identified two molecular mechanisms that could explain the effect of nutrition on spermatogenesis and germ cell apoptosis in the adult male: 1) nutrition-induced changes in the expression of mRNAs in sheep testis, the functions of the differentially expressed mRNAs are mainly related to spermatogenesis and germ cell apoptosis, an important regulator in these processes are the regulatory relationships between miRNAs and mRNAs; 2) nutritional treatment causes differences in pre-mRNA alternative splicing, and these changes are closely involved in spermatogenesis and germ cell apoptosis in testis. Some differentially spliced genes (*CREM and DDX4*), and testis phenotype related genes (*CFLAR*, *PTPRC*, *F2R*, *MAP3K1*, *EPHA7*, *APP* and *BCAP31*) should be able to work as potential biomarkers for spermatogenesis and apoptosis. To make the study move forward, confirming the predicted functions of these genes using *in vivo* and *in vitro* experiments are required.

## Additional files

Additional file 1: Table S1. Phenotypic traits of sheep testes used for WGCNA analysis. “H” indicates sheep fed with high diet, “L” indicates sheep fed with low diet. P1 indicates testis weight (g), P2 indicates sperm number per testis, P3 indicates diameter of seminiferous tubule, P4 indicates volume of seminiferous epithelium (x 1012 μm3), P5 indicates change of scrotal circumference (cm) and P6 indicates apoptotic germ cells/tubule. (XLSX 10 kb)
Additional file 2: Figure S1. Transcription profiles plotted across the sheep genome, showing the distribution of the RNA-seq read density along the length of each chromosome. Each vertical blue line represents log2 of the frequency of reads plotted against the chromosome coordinates. (PDF 880 kb)
Additional file 3: Table S2. Differentially expressed mRNAs in testis from sheep fed a low or high diet (N = 8 for each treatment). Note: Fold change (FC) = CPM of low diet group/CPM of high diet group. CPM (Counts per million) = (mRNAs reads number/total reads number per library) × 1,000,000. The significant DE mRNAs were determined by false discovery rate (FDR) < 0.05. (XLSX 168 kb)
Additional file 4: Table S3. The 10 most related function clusters of differentially expressed mRNAs by DAVID analysis. The lower p value indicates greater relevance. (XLSX 12 kb)
Additional file 5: Figure S2. qRT-PCR validation of differentially expressed genes. mRNA expressions from qRT-PCR are shown by line graphs on the top and values are shown on the right Y-axis as relative expression (2-ΔΔCt). mRNA expressions from RNA-Seq are shown by bar graphs on the bottom and values are shown on the left Y-axis as log2 (normalised reads number). a, b - indicate the significant difference in the relative expression of mRNAs detected via qRT-PCR at *P*<0.05; A, B - indicate significant difference in the expression of mRNAs detected from RNA-seq at FDR <0.05. Data are presented as Mean±Standard deviation. (PDF 610 kb)
Additional file 6: Table S4. Identification of putative miRNA-mRNA pairs on the basis of target prediction and the negative regulatory effect of miRNAs on mRNA expression levels. (XLSX 26 kb)
Additional file 7: Figure S3. Regulatory relationships for two pairs of miRNAs and mRNAs that were differentially expressed in sheep testis following nutritional treatment: oar-novel-miR-33 with 68 mRNAs, and oar-novel-miR-31 with 52 mRNAs. (PDF 937 kb)
Additional file 8: Table S5. Identification of alternative splicing events with Tophat2 software. (XLSX 1164 kb)
