# Supplementary material for: Functional changes in mRNA expression and alternative pre-mRNA splicing associated with the effects of nutrition on apoptosis and spermatogenesis in the adult testis
Source: BMC Genomics. 2017 Jan 10;18:64. doi: 10.1186/s12864-016-3385-8 (PMC5223305; doi:10.1186/s12864-016-3385-8)
Supplement: Additional file 2: Figure S1. — Transcription profiles plotted across the sheep genome, showing the distribution of the RNA-seq read density along the length of each chromosome. Each vertical blue line represents log2 of the frequency of reads plotted against the chromosome coordinates. (PDF 880 kb) [file 12864_2016_3385_MOESM2_ESM.pdf]

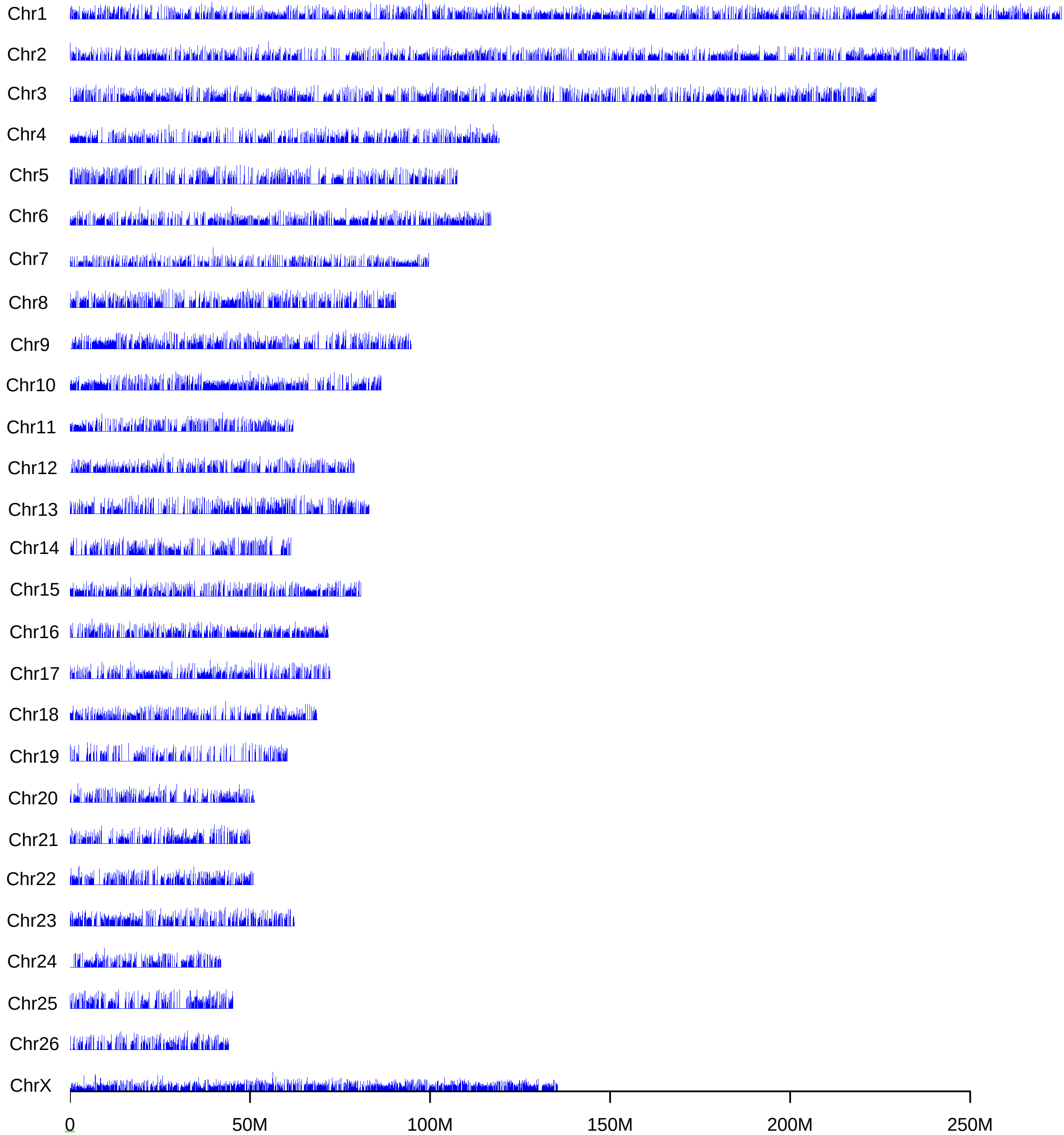
Chr1
Chr2
Chr3
Chr4
Chr5
Chr6
Chr7
Chr8
Chr9
Chr10
Chr11
Chr12
Chr13
Chr14
Chr15
Chr16
Chr17
Chr18
Chr19
Chr20
Chr21
Chr22
Chr23
Chr24
Chr25
Chr26
ChrX
0
50M
100M
150M
200M
250M

**Figure S1.** Transcription profiles plotted across the sheep genome, showing the distribution of the RNA-seq read density along the length of each chromosome. Each vertical blue line represents $\log_2$ of the frequency of reads plotted against the chromosome coordinates.
