# Supplementary material for: Functional changes in mRNA expression and alternative pre-mRNA splicing associated with the effects of nutrition on apoptosis and spermatogenesis in the adult testis
Source: BMC Genomics. 2017 Jan 10;18:64. doi: 10.1186/s12864-016-3385-8 (PMC5223305; doi:10.1186/s12864-016-3385-8)
Supplement: Additional file 5: Figure S2. — qRT-PCR validation of differentially expressed genes. mRNA expressions from qRT-PCR are shown by line graphs on the top and values are shown on the right Y-axis as relative expression (2-ΔΔCt). mRNA expressions from RNA-Seq are shown by bar graphs on the bottom and values are shown on the left Y-axis as log2 (normalised reads number). a, b - indicate the significant difference in the relative expression of mRNAs detected via qRT-PCR at P<0.05; A, B - indicate significant difference in the expression of mRNAs detected from RNA-seq at FDR <0.05. Data are presented as Mean±Standard deviation. (PDF 610 kb) [file 12864_2016_3385_MOESM5_ESM.pdf]

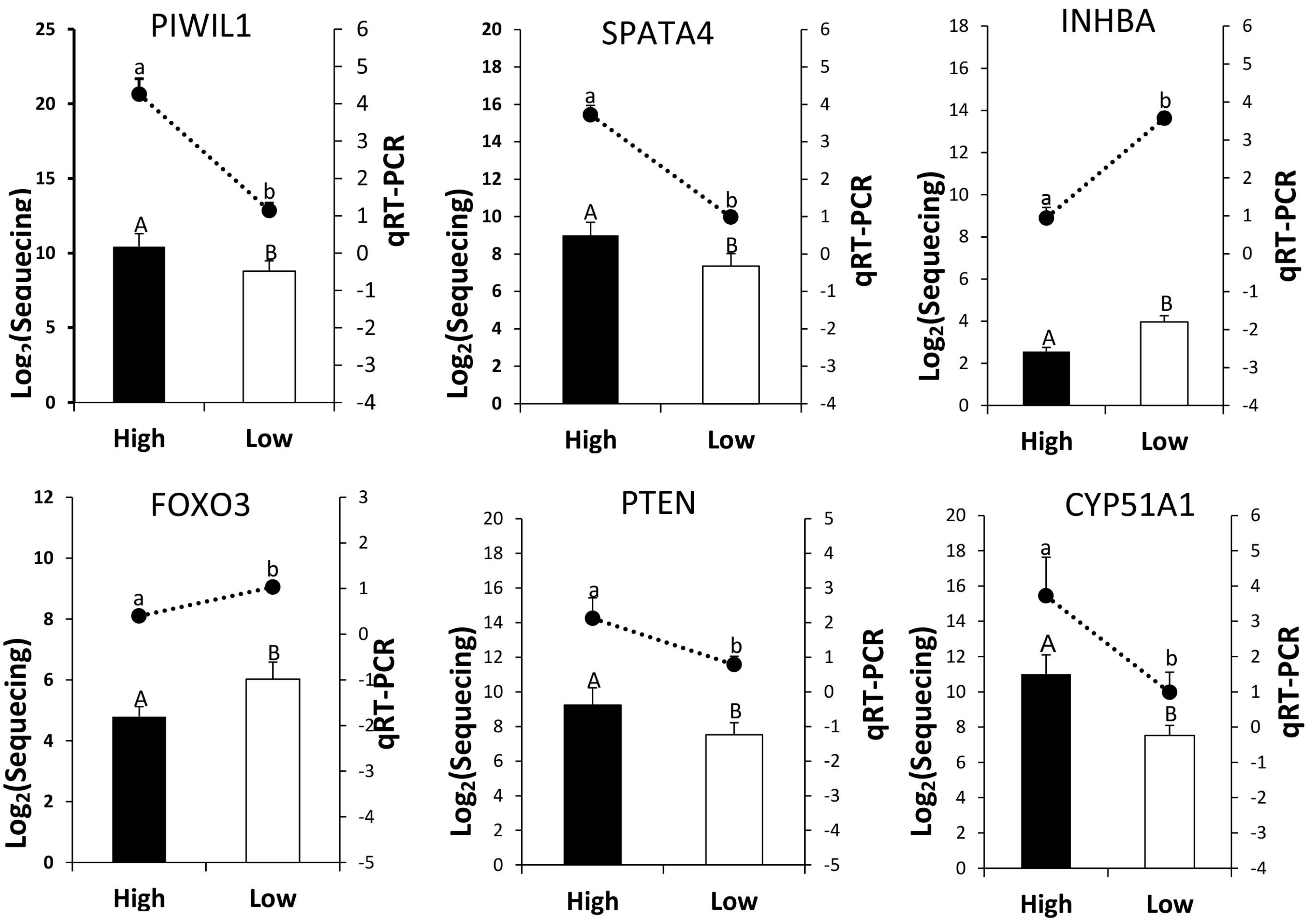
PIWIL1
SPATA4
INHBA
FOXO3
PTEN
CYP51A1
Log2(Sequecing)
qRT-PCR
High
Low
a
b
A
B

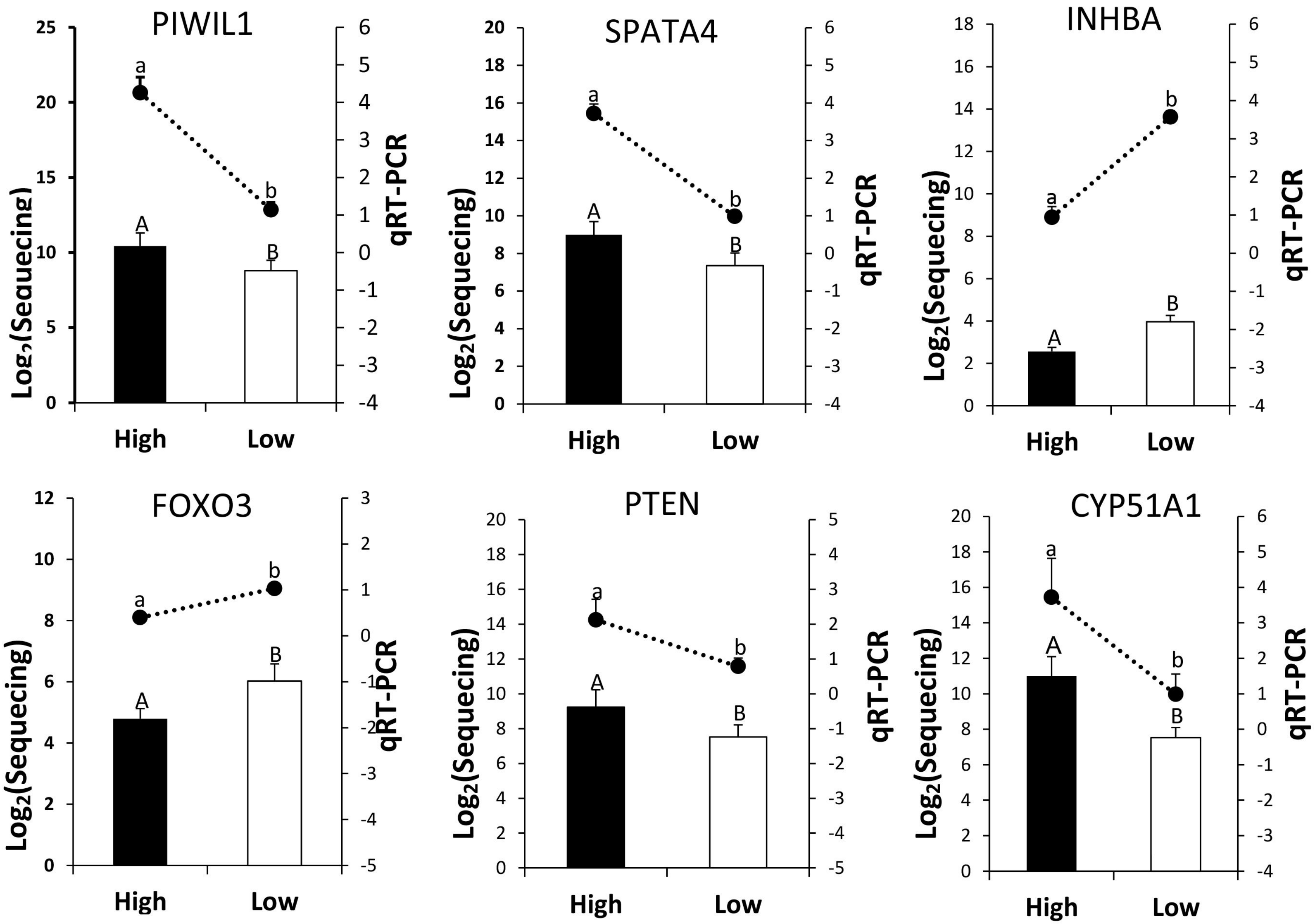
PIWIL1
SPATA4
INHBA
FOXO3
PTEN
CYP51A1
Log2(Sequecing)
Log2(Sequecing)
Log2(Sequecing)
Log2(Sequecing)
Log2(Sequecing)
Log2(Sequecing)
qRT-PCR
qRT-PCR
qRT-PCR
qRT-PCR
qRT-PCR
qRT-PCR
High
Low
High
Low
High
Low
High
Low
High
Low
High
Low

Figure S2. qRT-PCR validation of differentially expressed genes. mRNA expressions from qRT-PCR are shown by line graphs on the top and values are shown on the right Y-axis as relative expression (2-ΔΔCt). mRNA expressions from RNA-Seq are shown by bar graphs on the bottom and values are shown on the left Y-axis as log2 (normalised reads number). a, b - indicate the significant difference in the relative expression of mRNAs detected via qRT-PCR at $P<0.05$; A, B - indicate significant difference in the expression of mRNAs detected from RNA-seq at FDR <0.05. Data are presented as Mean±Standard deviation.
