# Supplementary material for: Functional changes in mRNA expression and alternative pre-mRNA splicing associated with the effects of nutrition on apoptosis and spermatogenesis in the adult testis
Source: BMC Genomics. 2017 Jan 10;18:64. doi: 10.1186/s12864-016-3385-8 (PMC5223305; doi:10.1186/s12864-016-3385-8)
Supplement: Additional file 7: Figure S3. — Regulatory relationships for two pairs of miRNAs and mRNAs that were differentially expressed in sheep testis following nutritional treatment: oar-novel-miR-33 with 68 mRNAs, and oar-novel-miR-31 with 52 mRNAs. (PDF 937 kb) [file 12864_2016_3385_MOESM7_ESM.pdf]

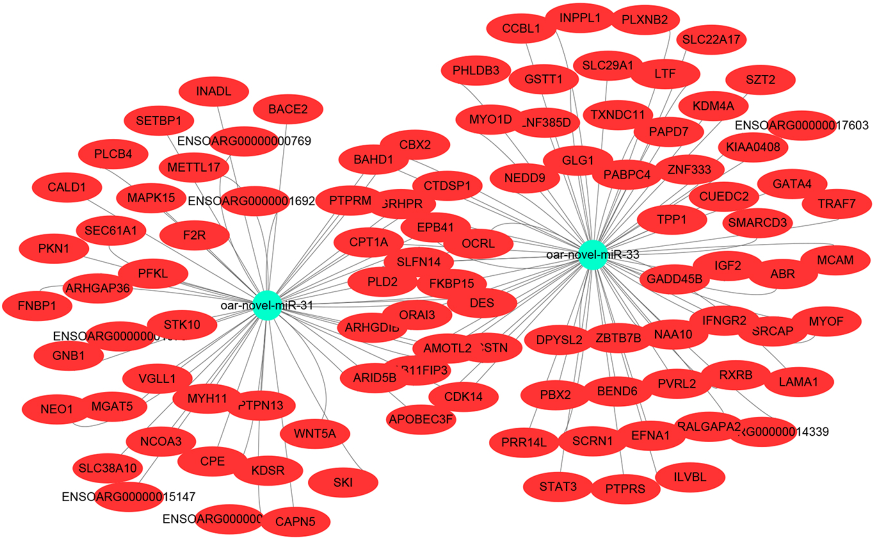
oar-novel-miR-31
oar-novel-miR-33
INADL
BACE2
SETBP1
ENSOARG00000000769
PLCB4
METTL17
CALD1
MAPK15
ENSOARG0000001692
SEC61A1
F2R
PKN1
PFKL
ARHGAP36
FNBP1
STK10
GNB1
VGLL1
MYH11
PTPN13
NEO1
MGAT5
NCOA3
WNT5A
CPE
KDSR
SLC38A10
SKI
ENSOARG00000015147
CAPN5
CBX2
BAHD1
CTDSP1
PTPRM
EPB41
OCRL
CPT1A
SLFN14
PLD2
FKBP15
DES
ORAI3
AMOTL2
ARID5B
CDK14
APOBEC3F
CCBL1
INPPL1
PLXNB2
SLC22A17
PHLDB3
GSTT1
SLC29A1
LTF
SZT2
KDM4A
MYO1D
TXNDC11
PAPD7
ENSOARG00000017603
KIAA0408
GLG1
NEDD9
PABPC4
ZNF333
GATA4
CUEDC2
TRAF7
TPP1
SMARCD3
IGF2
MCAM
ABR
GADD45B
IFNGR2
MYOF
SRCAP
DPYSL2
ZBTB7B
NAA10
RXRB
LAMA1
PBX2
BEND6
PVRL2
RALGAPA2
PRR14L
SCRN1
EFNA1
STAT3
PTPRS
ILVBL

**Figure S3.** Regulatory relationships for two pairs of miRNAs and mRNAs that were differentially expressed in sheep testis following nutritional treatment: oar-novel-miR-33 with 68 mRNAs, and oar-novel-miR-31 with 52 mRNAs.
